# Free Insulin-like Growth Factor (IGF)-I in Children with PWS

**DOI:** 10.3390/jcm11051280

**Published:** 2022-02-26

**Authors:** Layla Damen, Melitza S. M. Elizabeth, Stephany H. Donze, Sjoerd A. A. van den Berg, Laura C. G. de Graaff, Anita C. S. Hokken-Koelega

**Affiliations:** 1 Dutch Growth Research Foundation, 3016 AH Rotterdam, The Netherlands; stephanydonze@gmail.com (S.H.D.); a.hokken@erasmusmc.nl (A.C.S.H.-K.); 2 Department of Pediatrics, Subdivision of Endocrinology, Erasmus MC University Medical Center-Sophia Children’s Hospital, 3015 CN Rotterdam, The Netherlands; 3 Dutch Center of Reference for Prader-Willi Syndrome, 3015 GD Rotterdam, The Netherlands; l.degraaff@erasmusmc.nl; 4 Academic Center for Growth Disorders, Erasmus MC University Medical Center, 3015 GD Rotterdam, The Netherlands; 5 Department of Internal Medicine, Division of Endocrinology, Erasmus MC University Medical Center, 3015 GD Rotterdam, The Netherlands; m.elizabeth@erasmusmc.nl (M.S.M.E.); s.a.a.vandenberg@erasmusmc.nl (S.A.A.v.d.B.); 6 Department of Clinical Chemistry, Erasmus MC University Medical Center, 3015 GD Rotterdam, The Netherlands

**Keywords:** free insulin-like growth factor I, immunoreactive insulin-like growth factor I, Prader–Willi syndrome, growth hormone

## Abstract

In children with Prader–Willi syndrome (PWS), the standard growth hormone (GH) dose often results in high immunoreactive IGF-I levels. These high immunoreactive IGF-I levels lead to concern because their long-term effects are unknown. As a result, clinicians have to lower the GH dose, which worsens body composition and quality of life. As clinical features do not seem to correspond to immunoreactive IGF-I values, it is questionable whether immunoreactive IGF-I is a suitable marker for GH dosing, or whether another parameter better reflects IGF-I bioavailability and bioactivity. We, therefore, investigate serum immunoreactive IGF-I, free IGF-I and IGFBP-3 levels in 70 GH-treated children with PWS. Our study showed that, although immunoreactive IGF-I levels were high (>2 SDS) in the vast majority of prepubertal and pubertal children, free IGF-I SDS levels were <0 SDS in most and <1 SDS in all. Free IGF-I correlated with the immunoreactive IGF-I, IGFBP-3 and IGF-I/IGFBP-3 ratio. We conclude that there is a major discrepancy between immunoreactive and free IGF-I levels. While in the majority of GH-treated children with PWS, immunoreactive IGF-I levels were high, free IGF-I levels were <0 SDS in most. Our data appear to be very reassuring and suggest that free IGF-I levels should also be taken into consideration when the immunoreactive IGF-I levels are >2 SDS in GH-treated children with PWS.

## 1. Introduction

Prader–Willi syndrome (PWS) is a rare multisystem genetic disorder caused by the lack of expression of paternally inherited imprinted genes on chromosome 15q11-q13 [1,2]. PWS is characterized by muscular hypotonia, short stature, abnormal body composition, developmental delay, behavioral problems and hyperphagia, which can result in severe obesity when uncontrolled [2,3,4].

The benefits of growth hormone (GH) treatment in children are well established, as GH improves body composition, psychomotor development, cognition and linear growth [5,6,7,8,9]. However, in children with PWS, the treatment with a standard GH dose often results in high serum immunoreactive insulin-like growth factor (IGF)-I levels, with levels often being >2 SD score (SDS), which is a concern for clinicians treating children with PWS [5,10,11,12]. On the other hand, it is well known that children with PWS often require relatively high serum immunoreactive IGF-I levels to maintain an acceptable body composition with a fat mass percentage (FM%) at maximum 2 SDS [5,10].

In the circulation, IGF-I is mainly bound to insulin-like growth factor binding proteins (IGFBPs). The GH-dependent IGFBP-3 is the most important carrier protein of IGF-I in the circulation and accounts normally for more than 90% of IGF binding [13]. Under normal circumstances, only less than 1% of the total plasma IGF-I pool is unbound (‘free’), which is the biologically active form [14]. Currently, immunoreactive IGF-I levels (‘total IGF-I’) and not free IGF-I levels are used in routine clinical practice to titrate the GH dose.

Our previous study on IGF bioactivity showed that in children with PWS, most serum IGF-I is sequestered in the ternary 150-kDA complex and that there is a disrupted correlation between immunoreactive IGF-I SDS levels and IGF bioactivity [15]. This indicated that immunoreactive IGF-I levels are not appropriate for GH dosing in children with PWS. However, the IGF bioactivity assay is laborious and expensive. Another way to estimate biologically active IGF-I is measurement of serum free IGF-I. Currently, no studies have investigated free IGF-I levels in children with PWS.

We, therefore, compare free IGF-I levels with immunoreactive IGF-I levels in the serum of GH-treated children with PWS. We hypothesize that during GH treatment, free IGF-I would be in the normal range despite high immunoreactive IGF-I levels. Secondly, free IGF-I levels in children with PWS are compared with free IGF-I levels in age-matched healthy controls. To investigate if we could find a proxy for free IGF-I levels, correlations of free IGF-I with immunoreactive IGF-I, IGFBP-3 and the IGF-I/IGFBP-3 ratio are determined. In addition, we investigate the associations between free IGF-I and body composition.

## 2. Methods

### 2.1. Subjects

We included 70 children participating in the Dutch PWS Cohort study, a study evaluating the effects and safety of long-term GH treatment in children with PWS. Inclusion criteria for the current analysis were (1) genetically confirmed diagnosis of PWS by a positive methylation test; (2) receiving GH treatment for at least 1 year; and (3) having serum available for measurement of immunoreactive IGF-I, IGFBP-3 and free IGF-I. The group was divided in a prepubertal PWS group (girls with Tanner stage M1 and boys with testes volume < 4 mL) and a pubertal PWS group (girls with Tanner stage ≥ M2 and boys with testes volume ≥ 4 mL).

The control group consisted of 272 healthy, age and sex-matched controls. Their blood samples were collected at the STAR medical center in The Netherlands. They visited the center for a routine check-up and had neither a growth problem nor an acute or chronic disease.

The study protocol of the Dutch PWS Cohort study was approved by the Medical Ethics Committee of the Erasmus University Medical Center. Written informed consent was obtained from parents and children older than 12 years; assent was obtained in children younger than 12 years of age. For the inclusion of healthy controls ethical review and approval were waived by the Medical Ethics Committee of the Erasmus University Medical Center.

### 2.2. Anthropometric Measurements and Body Composition

Children with PWS were treated with GH in a standard dose of 1 mg/m^2^/day. They were examined every 3 months by the PWS Team of the Dutch Growth Research Foundation in collaboration with pediatric endocrinologists and pediatricians. At each visit, the GH dose was adjusted to the calculated body surface area. In case serum immunoreactive IGF-I levels increased above >3 SDS, the GH dose was lowered [15].

Standing height and weight were measured as described previously [15]. Height, weight and body mass index (BMI) SDS were calculated with Growth Analyser 4.0 (available at www.growthanalyser.org) and were adjusted for gender and age according to Dutch reference values [16,17].

Fat mass (FM) and lean body mass (LBM) were measured by Dual-energy X-ray Absorptiometry (DXA) (Lunar Prodigy; GE Healthcare), as previously described [15].

### 2.3. Assays

After overnight fasting, blood samples were collected for the assessment of immunoreactive IGF-I, IGFBP-3 and free IGF-I. After centrifugation, serum samples were immediately frozen and stored at −80 °C until assayed. Measurements of serum immunoreactive IGF-I and IGFBP-3 were performed at the Endocrine laboratory of the Erasmus University Medical Center using the IDS-iSYS (Immunodiagnostic Systems, IDS, Boldon Colliery, UK), with an interassay CV of <6% and <5.1%, respectively. Levels of immunoreactive IGF-I were expressed as SDS, adjusting for age and gender [18].

Free IGF-I was measured manually using the AnshLabs Free IGF-I kit (AL-122, AnshLabs, Houston, TX, USA). The assay employs a two-site antibody principle to measure the dissociable fraction of IGF-I. Samples from PWS patients and controls were randomized and measured in duplicate to minimize plate-to-plate bias.

The IGF-I/IGFBP-3 ratio was calculated, as the molar ratio between immunoreactive IGF-I and IGFBP-3. IGF-I/IGFBP-3 ratio has been suggested to reflect an estimate of tissue available IGF-I [19].

### 2.4. Statistics

Statistical analysis was performed with SPS 24.0. For free IGF-I, individual SD-scores were calculated using the following formula: SD-score (x – average x/SD), where x is the actual free IGF-I level of the individual, average x is the average free IGF-I level of healthy controls, and SD is SD for the mean of healthy controls. Results were expressed as median (interquartile range, IQR). Data were analyzed for the prepubertal and pubertal children separately. Mann–Whitney U test was used to calculate differences between the prepubertal and pubertal groups. One-sample Wilcoxon signed rank test was used to compare the median of PWS patients with the median of healthy controls. Correlations were calculated with non-parametric bivariate correlations and expressed by Spearman’s rho to assess relationships between free IGF-I and serum levels of immunoreactive IGF-I, IGFBP-3, IGF-I/IGFBP-3 ratio and body composition.

## 3. Results

### 3.1. Clinical Characteristics

The prepubertal PWS group consisted of 40 children and the pubertal group of 30 children. The clinical characteristics of both groups are shown in Table 1. The median (IQR) age in the prepubertal group was 6.7 (5.6–9.0) years and median (IQR) age in the pubertal group was 14.2 (12.1 to 15.4) years. The prepubertal group started GH treatment at a median age of 1.3 (0.8 to 2.0) years, which was significantly younger than the pubertal group who started at a median age of 3.9 (2.2 to 6.0) years, *p* < 0.001. The median (IQR) GH dose was similar for the prepubertal and pubertal group (1.0 (0.7 to 1.0) mg/m^2^/day and 1.0 (0.8 to 1.0) mg/m^2^/day, respectively, *p* = 0.20).

### 3.2. Serum Free IGF-I and Immunoreactive IGF-I in Prepubertal and Pubertal Children with PWS

**Figure 1:**
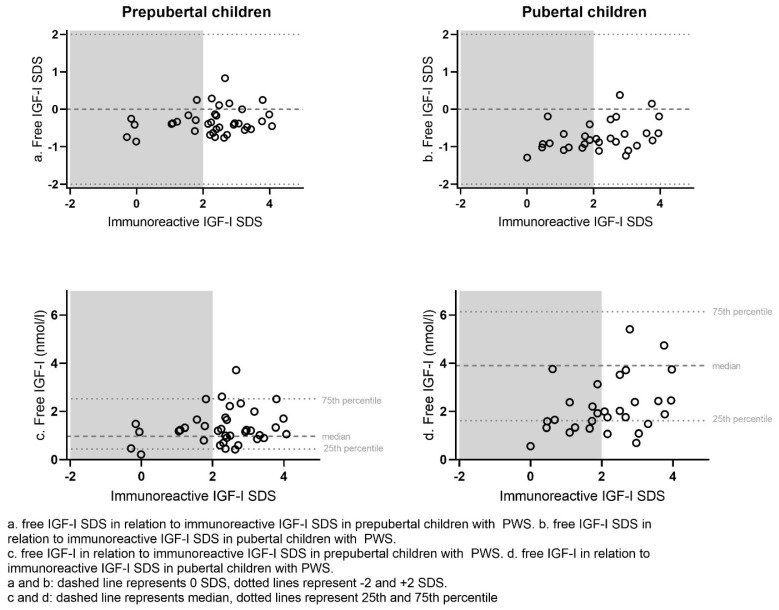
Free IGF-I and free IGF-I SDS in relation to immunoreactive IGF-I SDS in prepubertal and pubertal children with PWS.

Figure 1 shows free IGF-I and free IGF-I SDS in relation to immunoreactive IGF-I SDS. In the prepubertal children with PWS, 28 (70.0%) of children had an immunoreactive IGF-I SDS > 2 SDS, of which 9 (22.5% of the prepubertal group) had an immunoreactive IGF-I SDS > 3 SDS (Table 2). In the pubertal group, 17 (56.7%) children had an immunoreactive IGF-I SDS > 2 SDS, of which 7 (23.3% of the pubertal group) had an immunoreactive IGF-I SDS > 3 SDS. On the contrary, none of the prepubertal and pubertal children had a free IGF-I > 2 SDS. Only six (15.0%) prepubertal and two (6.7%) pubertal children with PWS had a free IGF-I > 0 SDS, while none of them had a free IGF-I > 1 SDS (Table 2).

The median (IQR) free IGF-I was 1.20 (0.86 to 1.66) nmol/L in the GH-treated prepubertal children with PWS, which was significantly lower than the median free IGF-I of 1.90 (1.33 to 2.62) nmol/L in the GH-treated pubertal children with PWS (*p* < 0.001) (Table 2). However, median free IGF-I SDS was −0.4 (−0.6 to −0.2) SDS in the prepubertal children, which was significantly higher than the median free IGF-I SDS of −0.8 (−1.0 to −0.6) in the pubertal children (*p* < 0.001). The median (IQR) immunoreactive IGF-I SDS was similar in the prepubertal and pubertal PWS children (2.4 (1.8 to 2.9) SDS vs. 2.2 (1.2 to 3.0) SDS, *p* = 0.66). The ratio of IGF-I/IGFBP-3 was significantly lower in the prepubertal group than in the pubertal group (0.23 (0.19 to 0.26) versus 0.32 (0.27 to 0.36) resp. *p* < 0.001). There were no significant differences in any of the parameters between boys or girls.

### 3.3. Serum Free IGF-I Levels in GH-Treated Children with PWS Compared to Healthy Controls

**Figure 2:**
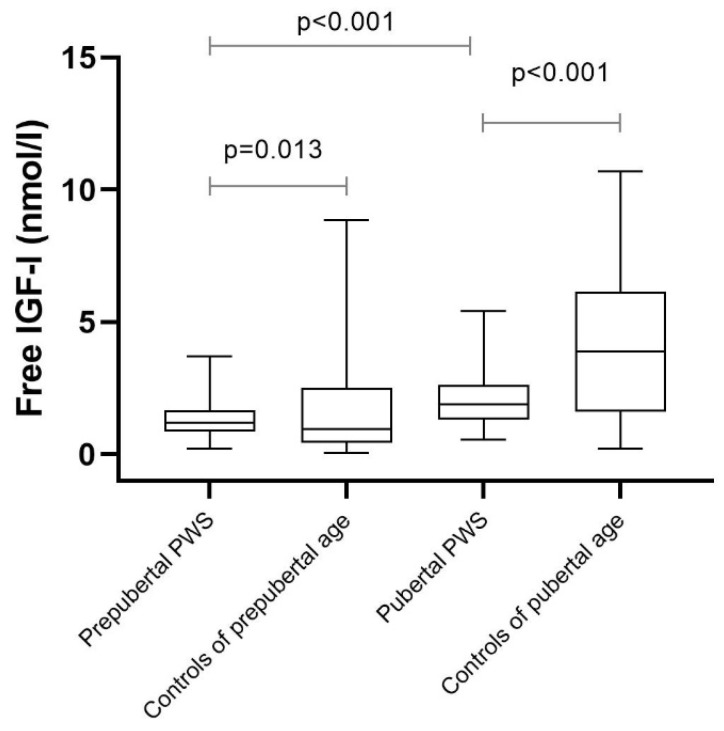
Free IGF-I in GH-treated prepubertal and pubertal children with PWS and healthy controls.

Figure 2 shows median free IGF-I levels in prepubertal and pubertal children with PWS in comparison with healthy controls. The median (IQR) free IGF-I was 1.20 (0.86 to 1.66) nmol/L in prepubertal children with PWS, which was significantly higher than the median of 0.97 (0.45 to 2.52) nmol/L in healthy controls (*p* = 0.013). In pubertal children with PWS, the median (IQR) free IGF-I was 1.90 (1.33 to 2.62) nmol/L, which was significantly lower than the median of 3.90 (1.61 to 6.14) in healthy controls (*p* < 0.001). The median (IQR) free IGF-I SDS in both prepubertal and pubertal children with PWS was significantly lower than the 0 SDS (*p* < 0.001), being −0.4 (−0.6 to −0.2) SDS and −0.8 (−1.0 to −0.6) SDS, respectively.

### 3.4. Correlations of Free IGF-I to Identify a Potential Proxy

**Figure 3:**
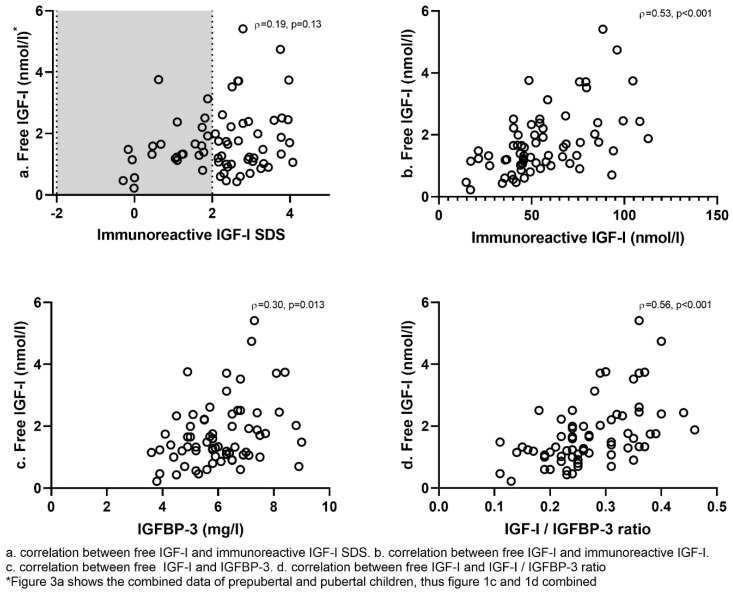
Correlations between free IGF-I and immunoreactive IGF-I, IGFBP-3 and IGF-I/IGFBP-3 ratio.

Figure 3 shows the correlations between free IGF-I and various parameters. In the total group, free IGF-I correlated significantly with immunoreactive IGF-I (rho = 0.53, *p* < 0.001), IGFBP-3 (rho = 0.30, *p* = 0.013) and strongest with the IGF-I/IGFBP-3 ratio (rho = 0.56, *p* < 0.001). Free IGF-I (nmol/L) did, however, not correlate with immunoreactive IGF-I SDS (rho = 0.19, *p* = 0.13). Free IGF-I SDS correlated only weakly with IGF-I SDS (rho = 0.26, *p* = 0.03). Free IGF-I correlated with GH dose in mg/m^2^/day (rho = 0.33, *p* = 0.006), but immunoreactive IGF-I only tended to correlate with the GH dose (rho = 0.23, *p* = 0.054). Neither free IGF-I nor immunoreactive IGF-I correlated with BMI SDS, FM% SDS or LBM SDS.

## 4. Discussion

This study shows that, while 70.0% of prepubertal children with PWS and 56.7% of pubertal children with PWS had a serum immunoreactive IGF-I > 2 SDS, free IGF-I was within the normal reference range in all children, which is reassuring. In fact, free IGF-I SDS was relatively low, as 85.0% of the prepubertal children and 93.3% of the pubertal children with PWS had a free IGF-I < 0 SDS.

High serum IGF-I levels during GH treatment are a major concern for clinicians treating children with PWS, particularly because the lowering of the GH dose results in a loss of the positive effects on body composition. In the current study, immunoreactive IGF-I SDS was >2 SDS in the majority of children with PWS. In contrast, free IGF-I SDS was <0 SDS in 85.0% of prepubertal and 93.3% of the pubertal children with PWS. All patients with an immunoreactive IGF-I > 2 SDS had free IGF-I levels within normal reference ranges. The median free IGF-I SDS in both prepubertal and pubertal children with PWS was significantly lower than in healthy controls.

The finding that high serum immunoreactive IGF-I levels correspond to low free IGF-I levels might explain why children with PWS require relatively high serum immunoreactive IGF-I levels to maintain an acceptable body composition [5,10]. This suggests that immunoreactive IGF-I levels are not appropriate for GH dosing in children with PWS. Unfortunately, we could not find a proxy for the free IGF-I levels and there are currently no data regarding free IGF-I levels as a marker of the long-term safety of GH treatment.

There are data that suggest that free IGF-I might represent IGF bioactivity. An example is a study in adults with a PAPP-A2 mutation (metalloproteinase pregnancy-associated plasma protein A2). PAPPA-A2 liberates IGF-I from IGFBP-3 and IGFBP-5 and, as a consequence, patients with PAPP-A2 mutation have low free IGF-I levels. The patients with PAPP-A2 mutations showed progressive growth failure [20,21,22,23]. Whereas immunoreactive IGF-I, IGFBP-3 and acid labile subunit (ALS) levels were high, free IGF-I levels and IGF bioactivity were decreased in these patients [20,21,22,23]. This suggests that free IGF-I is a measure of IGF bioactivity, while immunoreactive IGF-I is not. Our findings suggest that measuring serum free IGF-I levels could contribute to the decision of whether or not to change the GH dose when high immunoreactive IGF-I levels are found in a GH-treated child with PWS, particularly when our results are confirmed in another study. If our findings are confirmed, we suggest to only lower the GH dose if both immunoreactive IGF-I levels and free IGF-I levels are elevated.

Free IGF-I expressed as SDS correlated with immunoreactive IGF-I expressed as SDS, which is in accordance with a study by Frystyk et al. [24]. However, we found a major discrepancy between the SDS values of immunoreactive IGF-I and free IGF-I, as immunoreactive IGF-I SDS was 2 SDS higher.

This is in line with our previous study in children with PWS, where we also found an incongruence between IGF bioactivity and immunoreactive IGF-I levels [15]. Thus, IGF-I SDS does not reflect free IGF-I SDS in GH-treated children with PWS.

The molar ratio between immunoreactive IGF-I and IGFBP-3 has been suggested to reflect free IGF-I [19]. We, therefore, investigated if this was the case in GH-treated children with PWS. We indeed found a correlation between free IGF-I and IGF-I/IGFBP-3 ratio, in accordance with previous studies [24,25], but this correlation was insufficient to use the IGF-I/IGFBP-3 ratio as a proxy.

In our previous study, IGF bioactivity was higher in young children compared to older children with PWS [15]. However, in the current study, free IGF-I was higher in older children than in younger children. This difference might be explained due to the fact that the bioactivity assay is also influenced by IGF-II [15,26,27]. Free IGF-I measurements are not influenced by IGF-II. The higher free IGF-I levels in older children in the present study are in accordance with previous studies in healthy children, which also describe an increase in free IGF-I during puberty [14,20,28]. The fact that free IGF-I SDS was lower in older children with PWS, suggests that the pubertal increase in free IGF-I levels is lower in PWS than in healthy controls.

Previous studies have shown that free IGF-I levels are decreased in patients with growth hormone deficiency (GHD) and increase in GHD patients during GH treatment to levels higher than levels in healthy controls [14,29,30]. In healthy adults, the administration of GH results in a greater increase in free IGF-I than increase in immunoreactive IGF-I [31]. We found that in the majority of GH-treated children with PWS, immunoreactive IGF-I SDS increased >2 SDS, while none had a free IGF-I SDS > 1 SDS. It appears that this incongruence might also exist in other syndromes, as immunoreactive IGF-I > 2SDS in combination with free IGF-I < 2 SDS was also found in GH-treated girls with Turner syndrome [32]. Our findings are also in accordance with a recent study in small for gestational age (SGA) children, showing an immunoreactive IGF-I > 2 SDS in 68% of SGA children following 1 year of GH treatment, whereas only 15% had bioactive IGF-I levels slightly above normal reference values [26].

Our previous study in children with PWS found increased 150-kD complex formation, indicating that most of the IGF-I is sequestered by ALS and IGFBP-3, which might be an explanation for the discrepancy between immunoreactive IGF-I SDS and free IGF-I SDS [15]. Another explanation could be the short half-life of free IGF-I compared to the half-life of immunoreactive IGF-I [24]. However, the fact that the free IGF-I levels correlated with the GH dose, while immunoreactive IGF-I did not, contradicts this explanation. Future studies should further address which factors are responsible for the discrepancy.

We did not find correlations between either immunoreactive IGF-I or free IGF-I with body composition parameters. As all children with PWS had been on GH treatment for several years, we could not investigate the growth response or change in body composition after GH start in relation to free IGF-I or immunoreactive IGF-I levels.

A limitation of the current study is that we only investigated free IGF-I levels cross-sectionally and were not able to follow the course of free IGF-I levels over a longer period. In addition, we could not investigate changes in free IGF-I levels following adjustments in GH dose or investigate relations between changes in free IGF-I levels and changes in body composition.

## 5. Conclusions

We found a major discrepancy between serum immunoreactive IGF-I SDS and free IGF-I SDS, with immunoreactive IGF-I levels being 2 SDS higher. All children with immunoreactive IGF-I levels above the reference range had free IGF-I levels <1 SDS. Our findings show that immunoreactive IGF-I levels are not appropriate for GH dosing in children with PWS. The fact that we found relatively low free IGF-I levels in all children with high immunoreactive IGF-I levels is reassuring. If our findings are confirmed in future studies, we suggest that free IGF-I levels should also be taken into consideration in the decision of GH dose lowering when the immunoreactive IGF-I levels are >2 SDS.

## Figures and Tables

**Table 1:** Clinical Characteristics of PWS groups.

	Prepubertal PWS Group		Pubertal PWS Group		*p*-Value ^
Number (females)	40 (14)		30 (13)		
Genetic subtype					
Deletion/mUPD/ICD/translocation/#	20/18/1/0/1		12/16/0/0/2		
Age at inclusion (yrs)	6.7	(5.6 to 9.0)	14.2	(12.1 to 15.4)	<0.001
Age at GH start (yrs)	1.3	(0.8 to 2.0)	3.9	(2.2 to 6.0)	<0.001
Height (SDS)	0.4	(−0.6 to 0.8)	−0.2	(−1.4 to 1.2)	0.62
BMI (kg/m^2^)	17.3	(16.0 to 20.7)	21.3	(19.3 to 23.0)	0.002
BMI for age (SDS)	0.8	(0.1 to 2.0)	1.0	(−0.3 to 1.6)	0.65
BMI for PWS (SDS)	−0.9	(−1.5 to 0.0)	−1.2	(−2.3 to −0.5)	0.07
Fat mass percentage (%)	36.1	(31.7 to 45.8)	39.2	(34.6 to 42.5)	0.51
Fat mass percentage (SDS) *	2.4	(2.0 to 2.9)	2.3	(2.0 to 2.6)	0.61
Lean body mass (SDS) *	−1.4	(−1.9 to −0.7)	−1.3	(−3.2 to −0.5)	0.69
GH dose (mg/m^2^/dag)	1.0	(0.7 to 1.0)	1.0	(0.8 to 1.0)	0.20

Data expressed as median (IQR), mUPD: maternal uniparental disomy. ICD: imprinting center defect. BMI: body mass index. #: unknown. GH: growth hormone. * Fat mass percentage, lean body mass and IGF-I SDS were calculated according to sex- and age-matched Dutch references. ^ *p*-value of the difference between prepubertal and pubertal group.

**Table 2:** Immunoreactive IGF-I, free IGF-I and IGFBP-3 levels in GH-treated children with PWS.

	Prepubertal PWS Group		Pubertal PWS Group		*p*-Value ^
Number	40		30		
Immunoreactive IGF-I (nmol/L)	44.0	(35.8 to 52.3)	68.9	(53.9 to 89.6)	<0.001
Immunoreactive IGF-I SDS	2.4	(1.8 to 2.9)	2.2	(1.2 to 3.0)	0.66
No of patients with IGF-I > 2 SDS * (%)	28	(70.0%)	17	(56.7%)	0.21
No of patients with IGF-I > 3 SDS * (%)	9	(22.5%)	7	(23.3%)	1.0
Free IGF-I (nmol/L) #	1.20	(0.86 to 1.66)	1.90	(1.33 to 2.62)	<0.001
Free IGF-I SDS	−0.4	(−0.6 to −0.2)	−0.8	(−1.0 to −0.6)	<0.001
No of patients with free IGF-I > 2 SDS (%)	0	(0%)	0	(0%)	
No of patients with free IGF-I > 1 SDS (%)	0	(0%)	0	(0%)	
No of patients with free IGF-I > 0 SDS (%)	6	(15.0%)	2	(6.7%)	0.45
IGFBP-3 (mg/L)	5.7	(4.7 to 6.5)	6.5	(5.8 to 7.4)	0.001
IGFBP-3 (nmol/L)	190.0	(156.7 to 216.6)	216.6	(193.3 to 246.6)	0.001
Molar ratio IGF-I/BP3	0.23	(0.19 to 0.26)	0.32	(0.27 to 0.36)	<0.001

Data are presented as median (IQR). * SDS according to age-and sex-matched reference values (26). # Median (IQR) free IGF-I in prepubertal healthy controls 0.97 (0.45 to 2.52) nmol/L and in pubertal healthy controls 3.90 (1.61 to 6.14) nmol/L. ^ *p*-value of the difference between prepubertal and pubertal group.

## Data Availability

Restrictions apply to the availability of some or all data generated or analyzed during this study to preserve patient confidentiality or because they were used under license. The corresponding author will on request detail the restrictions and any conditions under which access to some data may be provided.
